# Methods for Improving Aptamer Binding Affinity

**DOI:** 10.3390/molecules21040421

**Published:** 2016-03-28

**Authors:** Hijiri Hasegawa, Nasa Savory, Koichi Abe, Kazunori Ikebukuro

**Affiliations:** Department of Biotechnology and Life Science, Graduate School of Engineering, Tokyo University of Agriculture and Technology, 2-24-16 Naka-cho, Koganei, Tokyo 184-8588, Japan; s149383s@st.go.tuat.ac.jp (H.H.); nasa.savoury@gmail.com (N.S.); abe79kou@gmail.com (K.A.)

**Keywords:** aptamer, binding affinity, aptamer sequence, unnatural nucleotide, multivalent binding

## Abstract

Aptamers are single stranded oligonucleotides that bind a wide range of biological targets. Although aptamers can be isolated from pools of random sequence oligonucleotides using affinity-based selection, aptamers with high affinities are not always obtained. Therefore, further refinement of aptamers is required to achieve desired binding affinities. The optimization of primary sequences and stabilization of aptamer conformations are the main approaches to refining the binding properties of aptamers. In particular, sequence optimization using combined *in silico* sequence recombinations and *in vitro* functional evaluations is effective for the improvement of binding affinities, however, the binding affinities of aptamers are limited by the low hydrophobicity of nucleic acids. Accordingly, introduction of hydrophobic moieties into aptamers expands the diversity of interactions between aptamers and targets. Moreover, construction of multivalent aptamers by connecting aptamers that recognize distinct epitopes is an attractive approach to substantial increases in binding affinity. In addition, binding affinities can be tuned by optimizing the scaffolds of multivalent constructs. In this review, we summarize the various techniques for improving the binding affinities of aptamers.

## 1. Introduction

Aptamers are DNA or RNA fragments that bind target molecules and are generally developed using the *in vitro* systematic evolution of ligands by exponential enrichment (SELEX) selection process [1,2]. SELEX involves simple separation steps of DNA or RNA that recognize target molecules from DNA or RNA libraries, and the repetition of these steps with a gradual increase in selection pressure leads to the isolation of aptamers with high affinity. Due to various advantages over antibodies, such as ease of chemical synthesis and modification, lower immunogenicity than antibodies, and the ability to refold after denaturation upon returning to the native condition, aptamers are versatile biomaterials that can be used as sensor elements, drugs, and drug delivery systems [3,4,5,6,7,8].

Regardless of the intended application, high target affinity is a critical requirement of aptamers. However, aptamers with high affinities are not always isolated by conventional SELEX. Numerous reports show various techniques for improving affinities of aptamers including the optimization of various conditions such as buffer, ions, pH, temperature, and we would like to introduce other works for improvement of aptamers apart from these optimization of the environmental conditions.

Limitations of the binding affinities of aptamers identified using SELEX reflect two procedural flaws. One is the limitation of molecular diversities in the initial library and the other is the loss of potential high affinity aptamers during polymerase chain reaction (PCR). DNA or RNA molecules in SELEX libraries usually comprise four natural nucleotides, whereas antibodies comprise up to twenty amino acids. Molecular diversity of SELEX starting libraries is very important to obtain aptamers with high affinity but, in starting libraries, the diversity of experimentally applicable oligonucleotide molecules is limited. Furthermore, aptamer structures are essential for their function, but may be adversely affected during PCR. DNA molecules that form stable structures resist PCR amplification, leading to the loss of some target binding sequences, and the resultant oligonucleotides do not always have the desirable binding affinities for targets. Modern SELEX usually includes emulsion PCR as a non-biased amplification method [9,10], but the molecular diversities of libraries are still limited in these methods. Hence, further optimization of sequences and stabilization of their conformations are often performed to improve the binding affinities of aptamers.

Another limitation of aptamer binding affinities relates to their oligonucleotide structures, which limit the variety of interactions between aptamers and target molecules. Because aptamers comprise hydrophilic oligonucleotides, hydrophobic interactions between aptamers and target molecules are limited. Hence, the addition of hydrophobic nucleotides to aptamers may transmute binding properties. In addition, aptamers bind to their targets with a part of the accessible surface area of their structures, and increase in the contact area between aptamers and targets would result in an increase in the binding affinities. Aptamers are usually smaller than 30-mers, even though they are selected from 30 to 60-mer random sequence libraries [11,12,13]. Therefore, use of the longer libraries would not be a good way to increase contact areas, and increasing the valence of aptamers by connecting aptamer epitopes may directly enhance binding affinities of aptamers.

In this review, first, we discuss the influences of aptamer sequences on their structures and binding affinities. Then, we describe about the approaches to improving binding affinities of aptamers. These approaches are classified as: (1) optimization of aptamer sequences; (2) stabilization of aptamer structures; (3) introduction of hydrophobic portions into aptamers; and (4) conjugation of binding motifs. In Section 3.1, we discuss the improvements of aptamer binding properties that can be achieved with sequence optimization. In Section 3.2, we argue that stabilization of aptamer structures contributes to binding affinities. In Section 3.3, we discuss influences of introduced unnatural hydrophobic nucleotides, and in Section 3.4 we summarize the current research concerning the binding properties of multivalent aptamers.

## 2. From Sequence to Function

The affinities of aptamers can be improved through sequence optimizations, and the understanding of sequence–activity relationships of existing aptamers can be used to improve the properties of other aptamers. Here, we will discuss the relationship between aptamer sequences, structures and their functions.

### 2.1. Sequence to Structure

Aptamers are selected from oligonucleotide libraries with 30–60-mer variable regions, however, the length of selected aptamers are generally less than 30-mer [11,12,13]. The determination of binding sites of aptamer sequences based on secondary structure predictions and sequence truncation are an effective way to improve binding affinities. Additionally, the secondary structures of aptamers can be predicted from their sequences [14]. We isolated a DNA aptamer against VEGF-165 [15], and secondary structure prediction revealed that this aptamer would form three stem-loop structures. Kaur *et al.* conducted a few truncations on the stem-loop regions to understand the significance of stem-loop regions, and one of the truncated aptamers showed a significant increase in the binding affinity for the target [16]. Shangguan and coworkers identified binding sites of selected aptamers by sequence truncation and mutation based on comparison of their predicted secondary structures [17,18]. These truncated and mutated sequences were found to have similar or better binding abilities.

Site-directed mutagenesis enables identification of important nucleotides that recognize targets but has poor efficiency and cost effectiveness. In contrast, microarray analyses have revealed numerous sequence–activity relationships. In comprehensive DNA microarray mutagenesis studies of IgE-binding aptamers, point mutations at most positions within IgE aptamer sequences readily decreased the binding ability of aptamers [19]. The predicted secondary structure of IgE aptamers was stem-loop, and the majority of the base pair-conserving double mutations of the stem region did not have a significant effect on the binding affinities. Similarly, Tome and coworkers measured dissociation constants of aptamers for GFP and its mutants using the Illumina platform [20]. As for the IgE aptamer, most single point mutants of the GFP aptamer had lower binding affinities than canonical aptamers [20]. Additionally, in the case of some GFP aptamers with lower affinity, single point mutants are predicted to produce substantially altered secondary structures, and two mutations within double mutants confounded each other’s effects [20]. This means that single point mutations are not independent from each other and the interactions between a GFP aptamer and its target depend upon an intricate structure dictated by its sequence. The microarray analysis revealed that most bases within IgE and GFP aptamers were essential for forming correct structures.

### 2.2. Structure to Function

Aptamers fold into unique structures that usually include stems and loops. These structures are central to target molecule recognition and any disruptions result in poor binding abilities. Some streptavidin binding DNA aptamers that were generated from different libraries by different laboratories have the same bulge-hairpin secondary structure motif [21]. Moreover, several nucleotides in the loop and bulge, which were critical for binding, were present in all high affinity sequences.

**Figure 1:**
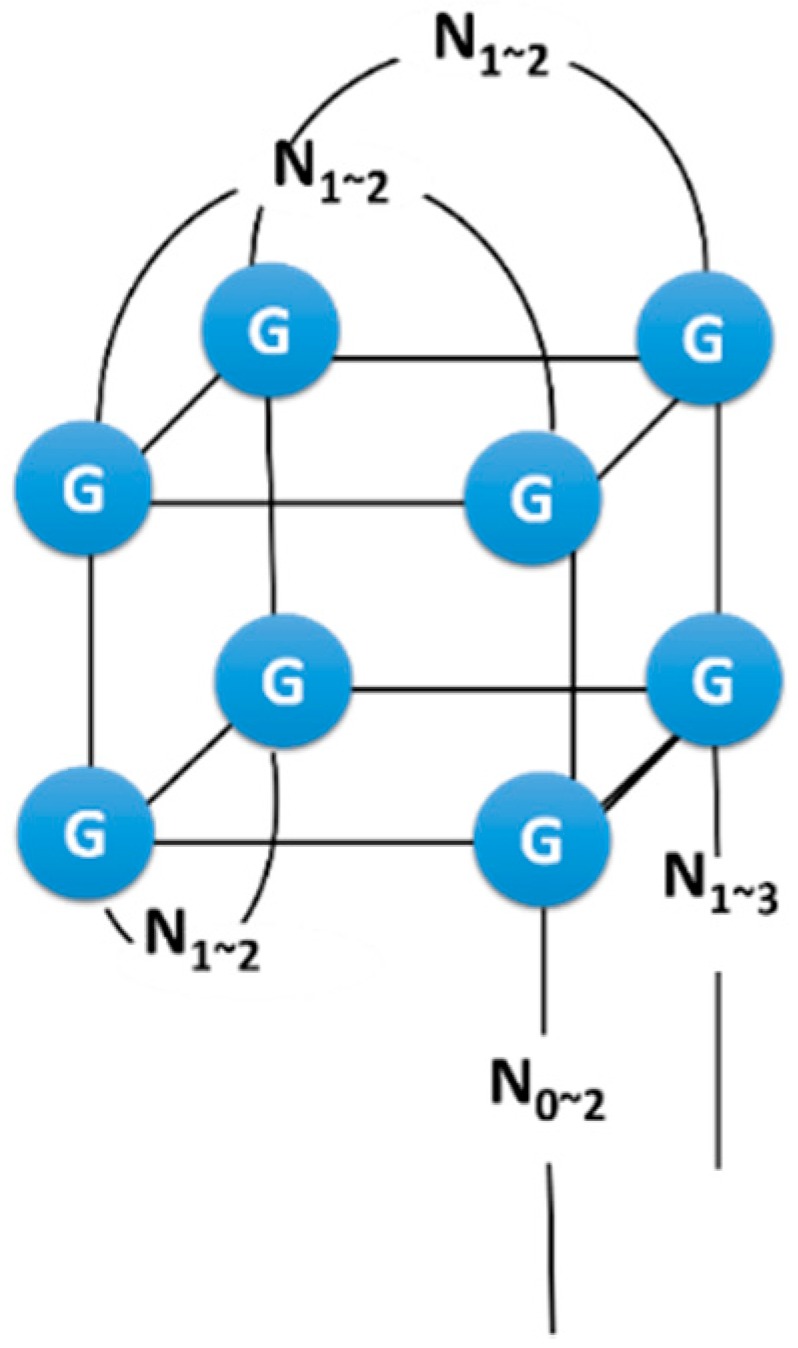
G-quadruplex structure of RNA aptamers for riboflavin.

The 2′OH group of RNA, which is an additional functional group in comparison with DNA, facilitates the formation of aptamer structures. Substitution of the ribose 2′H of DNA with a 2′OH led to thermal stabilization of some G-quadruplex (G4) structures [22]. G-quadruplexes are DNA and RNA structures formed from G-rich sequences. Hydrogen-bonded guanine-tetrad (G-quartet) is the basic structural motif of them [23], and they are formed by at least two stacked G-quartets. DNA homologs of RNA aptamers with G4 structures reportedly bind target molecules, although binding affinities of DNA homologs tend to be lower than those of RNA aptamers [24,25]. Specifically, the riboflavin binding RNA aptamer formed a G4 structure with two stacked G-quartet (Figure 1) and binding affinities of DNA homologs were weaker than those of RNA aptamers [25]. However, a DNA homolog with an additional guanosine in each G doublet could form a G4 structure with a core of three stacked G-quartet, reportedly bound to riboflavin with similar affinity to that of the RNA aptamer (two stacked G-quartet). The 2′OH group on these RNA aptamers likely contributes to stability of aptamer structures through hydrogen bonds, and destabilization of G4 structures with the loss of ribose 2′OH likely reduces binding affinity of DNA homologs. Conversely, additional planar G-quartets on the DNA homologs with two stacked G-quartet may stabilize the structure and improve target binding.

### 2.3. Example: ATP Aptamer

**Figure 2:**
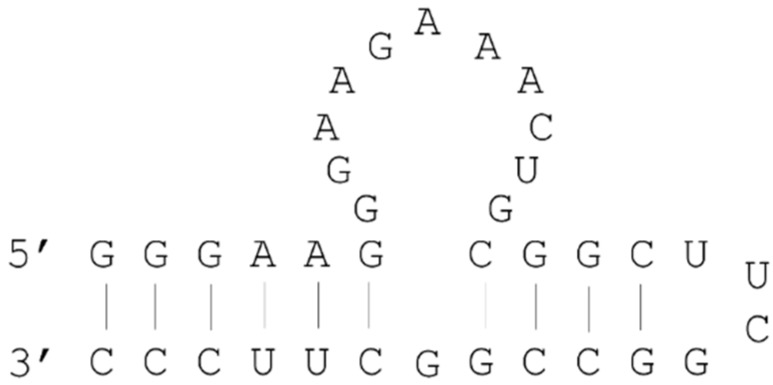
Secondary structure of the ATP-binding RNA aptamer.

Dieckmann *et al.* showed that alternations of 3D configurations of binding sites within ATP aptamers resulted in decreased binding affinity [26]. The secondary structures of ATP-binding RNA aptamers comprise an internal loop region and two stems flanking the loop (Figure 2); aptamer–ATP interactions occur in the loop [27,28]. Accordingly, deoxyribose nucleotide substitutions in stem regions greatly decreased the binding affinity for ATP, and the 3D structure formed by the A-form helix, which is a major structure of RNA duplexes, was shown to be essential for correct positioning of binding sites. In addition, experiments with single deoxyribose nucleotide substitutions within ATP binding RNA aptamers indicated that some 2′OH groups of this aptamer are essential for stability of the ATP–RNA aptamer binding structure [26].

## 3. Tuning of Aptamer Binding

### 3.1. Sequence Optimization

Aptamers are selected from huge libraries of random artificial oligonucleotides using SELEX, and after several rounds of selection, the enriched pool is cloned and sequenced. Following exclusion of redundant aptamers in the library, the sequences of potentially useful aptamers are obtained. Theoretically, whole sequence spaces can be searched from DNA libraries containing 24–25-mer randomized regions. Although *in vitro* combinatorial screening was established as the main strategy for aptamer development, with this approach it seems to be difficult to identify the best aptamers due to the limitation of library diversities [29] and artifacts such as PCR amplification biases [30,31].

The structures of aptamers are essential to recognize their target, however DNA molecules forming stable structures resist PCR amplification, leading to the loss of some target binding sequences. We tried to select an insulin-binding DNA aptamer from a DNA library which was expected to form various kinds of G4 structures because the insulin-linked polymorphic region (ILPR) oligonucleotide, which can be called a “natural” insulin-binding DNA aptamer, seemed to form a G4 structure [32]. The identified insulin-binding aptamers had a higher binding ability than the ILPR oligonucleotide, and the circular-dichroism spectrum measurement of the insulin-binding DNA aptamers indicated that the aptamers would fold into a G4 structure [32]. Davis *et al.* selected aptamers against GTP from partially structured RNA libraries containing a stable stem-loop [33]. Although they also used fully random libraries, the highest affinity aptamers had the engineered stem-loop. Although pre-structured SELEX resulted in identifying aptamers with high affinities, the selected sequences do not always have satisfactory binding affinities for target molecules. Thus further sequence refinement and optimization are usually required.

Microarray analyses have shown that the sequence-function space of oligonucleotides is a rugged landscape of local optima and minima [34]. Additionally, cost effective on-chip DNA synthesis has been used to facilitate analyses of DNA and target molecules. In particular, Asai *et al.* used on-chip DNA synthesis technology to obtain DNA aptamers for resorufin [35]. This selection method included on-chip selection and point-mutated sequences, and oligonucleotides for selection libraries were produced using genetic algorithms (GAs). Subsequently, binding of DNA and resorufin was evaluated using the DNA chip.

**Figure 3:**
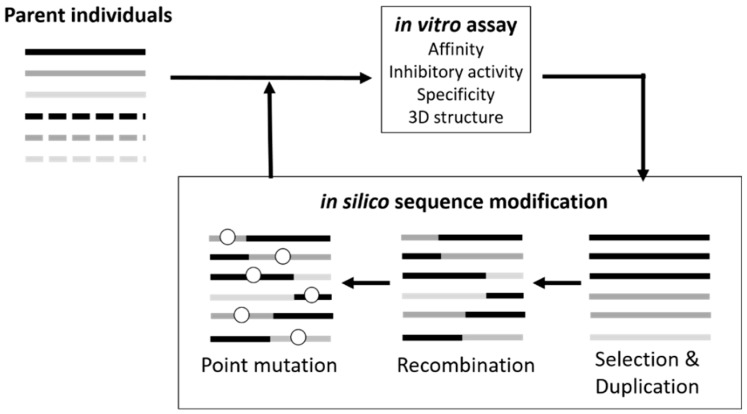
Scheme of *in silico* maturation.

To identify optimized aptamer sequences in rugged sequence spaces, we developed a genetic algorithm (GA)-based method designated *in silico* maturation (ISM) [36,37]. The process of GA-based strategies for optimizing aptamer sequences is, in general, an iterative cycle of *in silico* sequence modification, evaluation of fitness of each individual sequence, and selection of sequences with improve fitness [38]. ISM is a directed evolutionary technique employing an iterative process (Figure 3) of *in silico* sequence recombination followed by *in vitro* functional evaluation [37]. Although *in vitro* homogeneous recombination has been utilized to screen for improved aptamers [39], *in vitro* recombination of short oligonucleotides has very limited efficiency. However, *in silico* sequence recombination can be employed for any type of aptamer sequence, and we optimized aptamer sequences of G4 structures that inhibit thrombin and *Taq* DNA polymerase activities [36,40,41]. ISM was also employed to optimize loop-sequences of a three-way junction type aptamer against VEGF and a high affinity aptamer with a *K*_D_ of 52 nM was identified; subsequent multimerization achieved a *K*_D_ of 370 pM [42].

ISM-utilizing sequence recombination can also be used to find particular sequences or structures for aptamer binding from diverse parent sequences. In previous studies, we applied ISM to aptamers of various sequences selected by SELEX for prostate specific antigen (PSA) resulting in aptamer sequence convergence to a particular stem-loop structure [43]. ISM was also employed to specifically improve aptamers against *Proteus mirabilis* [44] and *Streptococcus mutans* [45]. Subsequently, 11 generations produced more than 300 sequences of aptamers for *S. mutans*, and these were improved by identifying core sequences that were required for binding. This schema search demonstrated the significance of the genetic algorithm in aptamer development. Hence, whereas ISM can target all functions of aptamers, including specificity and inhibitory activity to improve *in vitro* and *in vivo* evaluation processes, conventional SELEX approaches offer only affinity-based selection.

Aptamer development based on genetic algorithms was also demonstrated using high-throughput DNA microarrays [46]. Specifically, the closed loop aptameric directed evolution (CLADE) approach employs a genetic algorithm process starting with an array of random sequences, and explores the sequence–fitness landscape of aptamers. This approach was used to develop aptamers with affinity for fluorescent [46] and biotinylated proteins [47,48]. Hence, these high-throughput approaches using DNA microarrays and next generation sequencing may also offer a powerful strategy for aptamer sequence optimization.

### 3.2. Structure Manipulation

Stabilization of aptamer structures may improve their target binding affinities. Locked nucleic acid (LNA) is used to stabilize aptamer structures by virtue of a methylene bridge between the 2′-oxygen and 4′-carbon atoms of the ribose sugar and leads to greater rigidity than that of natural nucleotides. Although LNAs are synthetic nucleotides, they can form base pairs with natural nucleotides. Hence, incorporation of LNA nucleotides into DNA or RNA strands can increase the melting temperature of the duplex and stabilize nucleotide hybridization [49]. Accordingly, LNA modifications have been employed to stabilize duplexes within aptamers [50], and incorporation of LNA into double stranded regions of aptamers led to increased affinity constants [51,52]. In particular, systematic replacement of all A and G nucleotides in the sequence of an avidin-binding aptamer with LNA nucleotides led to an 8.5-fold enhancement in affinity [51]. Stabilizing aptamer structures reportedly improve binding abilities, however only modest differences between *K*_D_ values of canonical aptamers and modified aptamers have been reported.

### 3.3. Extended Alphabet (Artificial Nucleotides)

Aptamers against various target molecules have been generated using SELEX with unmodified libraries. However, the affinities of selected aptamers using conventional SELEX are not always high. One of the reasons for this would be that aptamers are hydrophilic molecules.

One approach to improve the affinities of aptamers against their targets is to increase the number of interactions between an aptamer and a target. Aptamer-target binding is generally mediated by polar, hydrogen bonding, and charge-charge interactions. In contrast, hydrophobic contacts that contribute to protein–protein interactions are limited. Hence, addition of functional groups that mimic amino acid side chains is warranted, and may expand chemical diversity [53] and enhance the binding affinity of aptamers. Additionally, since aptamers are hydrophilic molecules, we think water molecules would disturb the formation of interactions between aptamers and targets, not only hydrophobic interactions but also hydrogen bonding, charge-charge interactions.

**Figure 4:**
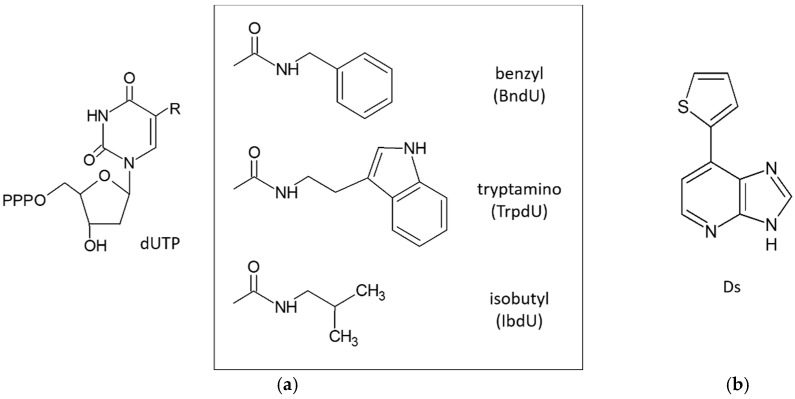
Chemical structures of the indicated bases. (**a**) Nucleotide triphosphate analogs modified at the 5-position (R) of uridine (dUTP): 5-benzylaminocarbonyl-dU (BndU); 5-tryptaminocarbonyl-dU (TrpdU); 5-isobutylaminocarbonyl-dU (iBudU); (**b**) 7-(2-thienyl)imidazo[4,5-*b*]pyridine (Ds).

However, SELEX with modified libraries is technically challenging because the modified nucleotides must be compatible with the enzymatic steps of SELEX. Nonetheless, Vaught *et al.* developed modified oligonucleotides that were compatible with the enzymology of SELEX [54]. Subsequently, Gold *et al.* tested the effects of modified nucleotide libraries according to these methods using thirteen human proteins that are difficult targets for unmodified libraries (these targets had repeatedly failed SELEX with unmodified libraries) [55]. The ensuing SELEX libraries were prepared with 2′-deoxyadenosine-5′-triphosphate (dATP), 2′-deoxyguanosine-5′-triphosphate (dGTP), 5-methyl-2′-deoxycytidine-5′-triphosphate (MedCTP), and one of three 2′-deoxyuridine-5′-triphosphate (dUTP) analogs, which were modified at the 5-position of dU with hydrophobic functional groups (5-benzylaminocarbonyl-dU (BndU), 5-tryptaminocarbonyl-dU (TrpdU) and 5-isobutylaminocarbonyl-dU (IbdU) (Figure 4a). Subsequently, SELEX with modified libraries resulted in high affinity aptamers with slow dissociation rates for all target proteins.

Kimoto *et al.* also reported the use of DNA libraries containing four natural nucleotides and an unnatural nucleotide carrying the hydrophobic base 7-(2-thienyl)imidazo[4,5-*b*]pyridine (Ds, Figure 4b) [56]. In addition to enhancing hydrophobicity, this library expanded the genetic alphabet to include unnatural nucleotides and the four natural nucleotides. In this study, DNA libraries containing oligonucleotides with approximately 1–3 Ds bases at predetermined positions were used because the oligonucleotides containing Ds could not be applied to conventional cloning and sequencing methods, which are required to isolate aptamers after selection. From this library, aptamers with Ds bases were identified for the human proteins vascular endothelial growth factor-165 (VEGF-165) and interferon-γ (INF-γ), and the affinities of these aptamers were substantially higher than those of aptamers comprising only natural bases. Moreover, these aptamers achieved binding specificity for targets. Hence, the introduction of hydrophobic Ds bases at a few positions increased both chemical and structural diversities of the DNA library, and led to isolation of DNA aptamers with desired binding affinities.

The Structure of a complex of a modified aptamer and a target protein has been reported [57]. A co-crystal structure of a modified aptamer bound to platelet-derived growth factor B (PDGF-BB) revealed that all hydrophobic nucleotides within the aptamer were in contact with the target, and most were clustered along the hydrophobic groove of PDGF. Furthermore, these hydrophobic functional groups contributed to unique intramolecular motifs that have not been observed in traditional aptamers. Hence, the introduction of hydrophobic nucleotides may expand the structural diversity of aptamers and the range of accessible protein targets. Accordingly, inserted hydrophobic nucleotides were used to recognize hydrophobic portions of target molecules, confirming that incorporation of hydrophobic nucleotides to libraries broadens the range of epitopes of aptamers, leading to the development of aptamer pairs.

Aptamers isolated from unmodified oligonucleotide libraries usually recognize positively charged target motifs. Hence, the epitopes of selected aptamers against a target tend to overlap, and aptamer pairs that recognize different epitopes within a target molecule are rarely isolated. However, Ochsner *et al.* recently isolated modified aptamer pairs that bind different epitopes of target cardiovascular risk marker proteins [58]. These aptamers were generated by a two-step SELEX procedure in which SELEX was initially performed using a free target and a modified random library, and a subsequent SELEX was performed with the target in complex with aptamer and a library containing various modified nucleotides.

SELEX with libraries containing modified nucleotides limits the efficiency of some processes, such as PCR amplification and cloning. Hence, further refinement is required for this approach, potentially using genetic algorithm based and other strategies that allow optimization of modifications and positions of aptamers with unnatural nucleotides, and techniques that reveal the effects of modified oligonucleotides on aptamer binding.

### 3.4. Joining of Binding Motifs

Joining of binding motifs of aptamers would result in increased contact areas between aptamers and target molecules, and the improvements of binding affinities of aptamers. Hence, multivalent interactions produce higher binding affinities than their corresponding monovalent interactions. Accordingly, we designed bivalent constructs [59] by connecting a 15-mer thrombin aptamer [11] and a 29-mer thrombin aptamer [13]. The ensuing *K*_D_ value of the bivalent construct was subnanomolar, and was less than 1/10 of those of the corresponding monovalent interactions. Subsequent binding kinetics studies showed that this enhancement of affinity primarily reflected much smaller *k*_off_ values.

**Figure 5:**
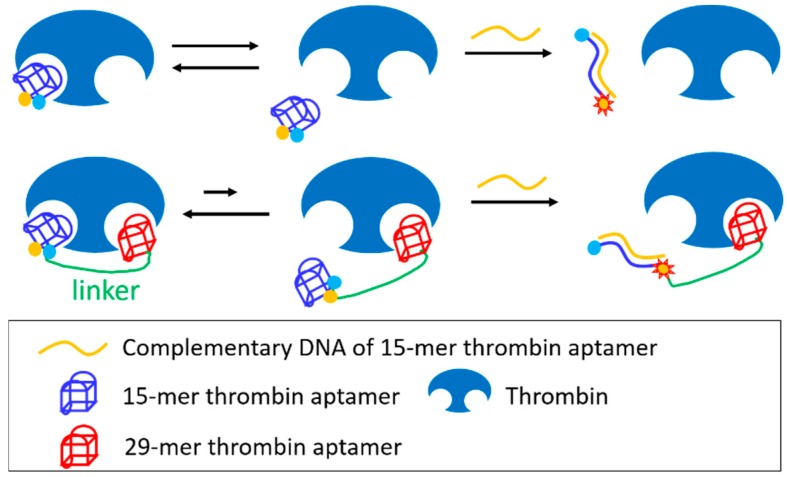
Cartoon to describe the *k*_off_ measurement of bivalent construct against thrombin.

The 15-mer thrombin aptamer binds to the fibrinogen binding exosite and inhibit thrombin activity, whereas the 29-mer thrombin aptamer binds the heparin binding site and had no inhibitory activity. In a study by Kim *et al.* the corresponding bivalent construct achieved better inhibition efficiency than the monovalent 15-mer aptamer [60]. These investigators then measured the binding kinetics of a 15-mer thrombin aptamer domain of the bivalent construct using a fluorescence resonance energy transfer (FRET) strategy (Figure 5), and showed that the *k*_off_ value of the 15-mer aptamer within the bivalent construct was slower than that of the monovalent aptamer, even though the *k*_on_ value was similar. Moreover, increased thrombin inhibition potency of the bivalent construct reflected a decreased *k*_off_ value due to cooperative binding.

**Figure 6:**
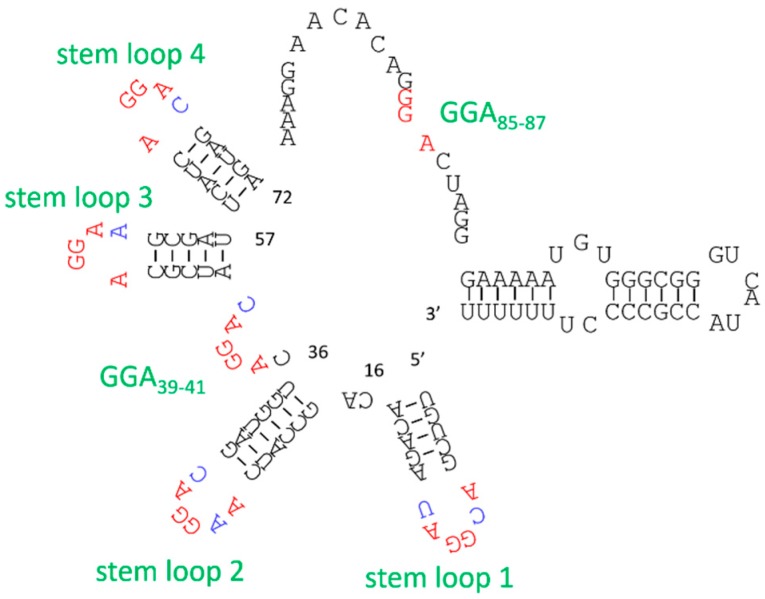
The predicted secondary structure of RsmZ sRNA. The conserved A(N)GGAX motif is shown in red, and the loop nucleotides N and X are shown in blue.

Multivalent interactions also occur between oligomeric proteins and various DNA and RNA sites in nature. As an example, the small ncRNA (sRNA) RsmZ was shown to trap the translation repressor protein CsrA/RsmE, and contained eight GGA motifs (Figure 6) that bind the RsmE protein dimer. Moreover, Duss *et al.* showed that three pairs of GGA motifs bind to RsmE as bivalent ligands [61]. Among these six GGA motifs that formed bivalent ligands, only one GGA motif bound RsmE with low-nM *K*_D_, and the others all had *K*_D_ values in the µM range. Moreover, these GGA motif pairs bound RsmE dimers sequentially and cooperatively, and their binding affinities were within the narrow range of 100–200 nM *K*_D_. Whereas the binding affinities of µM- *K*_D_ pairs were more than 10-fold greater than those of monovalent ligands, combinations of nM-*K*_D_ GGA motifs and µM-*K*_D_ GGA motifs produced lower affinity than expected based on binding affinities of individual ligands. However, shortening of the linker connecting the latter pair resulted in more than three-fold higher affinity for RsmE. These results indicate that the affinities of GGA motif pairs can be tuned by optimizing the scaffolds that join each binding site. Hence, the design of scaffolds that connect ligands is an important approach for producing multivalent constructs with high target affinity.

Binding affinity is thermodynamically related to the Gibbs free energy (ΔG = −*RTlnK*_a_ = ΔH − TΔS). Thus, when the linker of a multivalent construct is completely rigid and allows aptamer-target binding with a perfect fit, binding affinity could be maximally enhanced by entropic optimization. In contrast, small structural mismatches may lead to very weak binding affinity due to a negative impact on enthalpy [62]. Therefore, flexible linkers are usually applied because they can compensate for structural uncertainties. Accordingly, Tian *et al.* connected a 15-mer thrombin binding aptamer with a 29-mer thrombin binding aptamer using a polyethylene glycol (PEG) phosphoramidite linker, and showed that the binding affinity of this bivalent construct was 97-fold higher than those of the individual aptamers [63]. Mallikaratchy *et al.* also designed multivalent constructs for mIgM B-cell receptors using PEG phosphoramidite as a linker [52]. Whereas the monomeric aptamer against the mIgM B-cell receptor bound the target at 4 °C, it lacked affinity at physiological temperature. Hence, although bivalent constructs with PEG linkers that mimic the dimensions of antibodies had similar binding affinities as the corresponding monomers, the longer linker enabled bivalent aptamers to bind targets at physiological temperatures, whereas further elongation of the linker diminished binding affinity.

**Figure 7:**
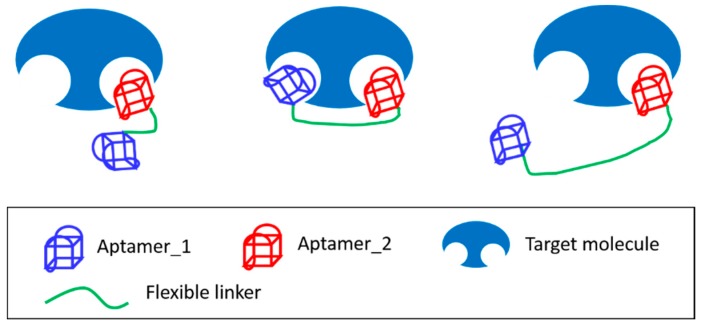
Diagrams of aptamer dimers binding to a target with distinct epitopes. Aptamers are shown with flexible linker whose length is too short (**left**), just right (**centre**), or longer (**right**) than necessary, to allow the ligands to span two binding sites.

In similar experiments, we evaluated the binding affinities of bivalent constructs for thrombin with flexible poly (dT) linkers of various lengths (0, 5, 10, 15, and 20 dTs) and selected the 5 dTs linker, which had the highest affinity for thrombin [59]. Subsequently, we showed that matching of the distance between ligands of multivalent constructs with those between binding sites increased affinity dramatically. Accordingly, when the persistent length of the flexible linker is much shorter than the distance between the two binding sites, the affinity of the multivalent ligand should equal the sum of the affinities of monovalent ligands (Figure 7, left) [64]. Hence, it follows that linkers with longer than suitable lengths will decrease effective concentrations (C_eff_) of the connected ligands, resulting in reduced binding affinity (Figure 7, right).

**Figure 8:**
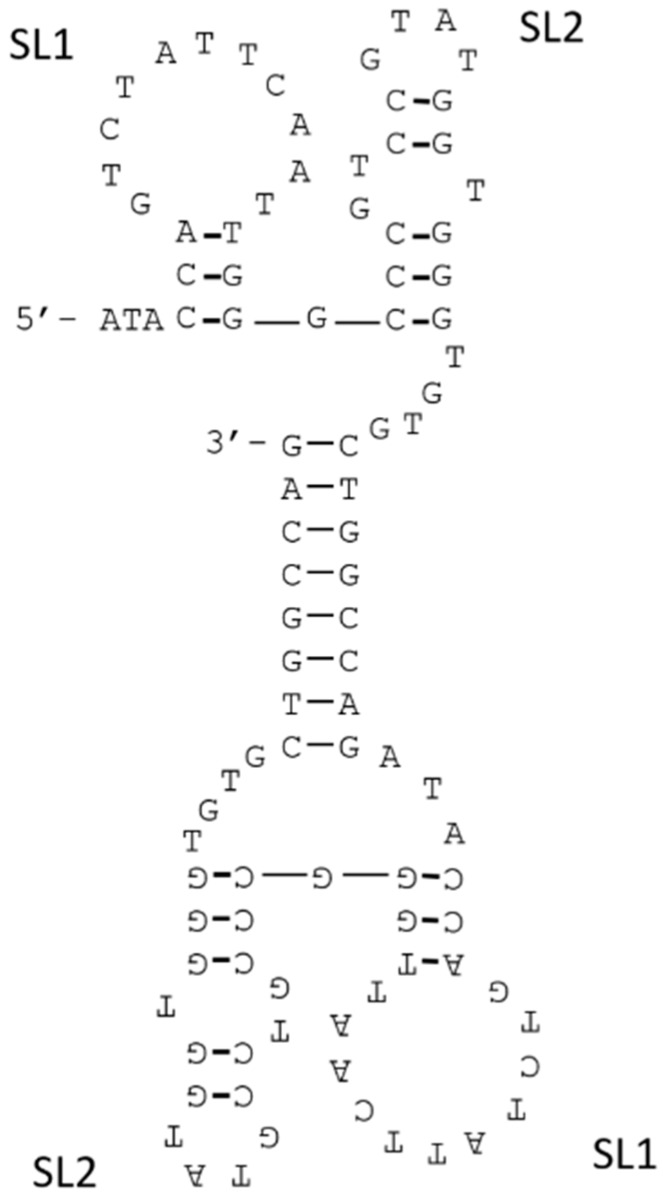
Secondary structure of the bivalent aptamer for VEGF-165, as predicted using the m-fold program [14].

In other studies, we isolated a DNA aptamer against VEGF-165 that had insufficient binding affinity for application [15]. Because VEGF-165 is a homodimer protein, we subsequently designed bivalent constructs by connecting two identical aptamers [59]. However, the distance between epitopes of aptamers was unclear, hence we connected the aptamers via 0, 10, 20 dTs linkers and compared those binding affinities to determine the most suitable length. And we used the truncated version of the VEGF-165 aptamer as a monomer aptamer. The affinities of aptamers for VEGF-165 were analyzed by surface plasmon resonance (SPR) measurement, and aptamer solutions were assayed on VEGF-165 immobilized sensor chips. The *K*_D_ values of aptamers were calculated by scatchard plot analysis which plots the SPR signal at equilibrium as a function of aptamer concentration. The scatchard plot of bivalent construct without a linker formed a straight line, and this bivalent construct had about 28-fold higher binding affinity (*K*_D_ = 17 nM) than that of the monovalent aptamer (*K*_D_ = 4.8 × 10^2^ nM). However, the plots of the bivalent constructs with the poly (dT) linkers did not form straight lines. These data indicate that the bivalent construct without linker would bind to the VEGF-165 with a 1:1 stoichiometry, but the bivalent constructs with poly (dT) linkers would not bind 1:1. The predicted secondary structure of the bivalent construct without a linker indicated the presence of a double stranded DNA (dsDNA) region (Figure 8), and the eight bases sequence at the 3′ end of the monomer aptamer was a palindrome that formed base-pairs in the bivalent construct. There were two stem-loops (SL1 and SL2) on both sides of the dsDNA, and Kaur *et al.* reported that SL2 would be important to bind to the VEGF-165 [16]. In the structure of the bivalent construct, the 8-bp dsDNA with 4-mer ssDNA flaking regions would become a linker and two SL2 sequences would bind to the two epitopes on VEGF-165. As stated above, flexible linkers can be used to avoid fatal effects on the enthalpy of aptamer–target complexes, although they have negative impacts on the intrinsic conformational entropy of the linker. The linker consisting dsDNA would avoid this conformational entropy loss, and addition of the short ssDNA region would be important to adjust the orientations of binding sites within the bivalent construct. Additionally, since the lengths of dsDNA regions could be identical, the binding sites at each end could stably bind to the VEGF-165, and the binding affinity of the bivalent construct was substantially improved.

The probability of finding the extended conformation of a flexible linker is low in solvent [62], and although the insertion of a rigid rod into the flexible linker may prohibit linker folding and present the ligand to the target with defined distance, most rod structures aggregate in water [65]. In contrast, DNA duplex architectures are soluble in water, and dsDNA behaves as a rigid rod [66,67]. Hence, dsRNA was used as a linker within a bivalent aptamer for heat shock factor 1 (HSF1), which forms a homotrimer [68]. The binding affinity of the ensuing bivalent construct, which carried a 12-bp dsRNA linker, was about 24-fold higher than that of the monomeric aptamer. Additionally, a bivalent construct with a 3-mer ssRNA sequence in the dsRNA region had 5-fold greater affinity than the original bivalent construct. Hence, scaffolds comprising a dsRNA region and a ssRNA region may simultaneously present the two aptamers with optimal distance and optimal orientation.

Ahmad *et al.* developed a bivalent construct with an ideal scaffold using a direct evolution strategy [69]. These investigators isolated bivalent aptamers comprising two thrombin aptamers joined by a 35-mer randomized region from a ssDNA library and showed approximately 200-fold higher affinity of the top binding bivalent aptamer than the 15-mer thrombin aptamer. Moreover, the predicted secondary structure of this bivalent aptamer indicated that most bases within the linker formed double-helical stems that were joined by a short single stranded region. These data demonstrate the utility of this evolution based approach for generating optimal multivalent constructs, and that this oligonucleotide linker for multivalent constructs is applicable to SELEX processes.

Multivalent interactions may be used to dramatically enhance binding affinities of aptamers to their targets. However, this approach only can be applied to aptamers that bind multivalent proteins or that recognize distinct epitopes of the same targets, and the ensuing aptamer pairs are rare. Recent reports demonstrate selection methods that enable isolation of aptamer pairs with activity against a single target [70,71,72]. In particular, we isolated an aptamer pair with activity against VEGF-165 using selection according to various VEGF-165 target domains, and the bivalent construct comprising these aptamers had higher binding affinity than each of the monomer aptamers alone [73]. More recently, an array-based approach was applied to isolation of aptamer pairs that recognize distinct epitopes on target proteins [74]. Subsequently, the identified aptamer pairs were conjugated with a flexible linker and high-affinity bivalent aptamers with a *K*_D_ of 97 pM had more than 200-fold greater binding affinity than either component aptamer. Hence, with increasing numbers of aptamer pairs, the effects of the multivalent approach to improving binding affinities may be promising.

## 4. Conclusions

Because aptamers with desired affinities are not always isolated by SELEX, refinements of selected aptamers are required to improve binding affinity. In our studies, the genetic algorithm- based method ISM enabled identification of optimized aptamer sequences in rugged functional sequence spaces. This approach is a powerful strategy for improving binding affinities of aptamers though sequence optimizations. Moreover, enhancements of binding affinities have been achieved through stabilization of aptamer structures. However, *K*_D_ values of aptamers with stable structures were only slight improvements on those of canonical aptamers. Hence, stabilization of aptamer structures may maintain binding properties in various environments, but offers little improvement in binding affinities. Among aptamers comprising natural nucleotides, hydrophobic interactions between aptamers and targets are limited. Thus, oligonucleotide libraries containing unnatural hydrophobic nucleotides have been used to isolate aptamers with hydrophobic moieties and activities against various proteins. Accordingly, introduction of hydrophobic moieties has expanded the repertoire of interactions and structural diversities of aptamers, and has led to substantial improvements in binding affinities. However, further refinements of SELEX with libraries containing modified nucleotides are required, and additional studies of specificities of aptamers containing hydrophobic moieties are warranted. Moreover, because most interactions between proteins are hydrophobic, non-specific binding between hydrophobic modified aptamers and proteins are a concern. Finally, multivalent aptamers form more interactions with target molecules than monomer aptamers, leading to substantial enhancements of binding affinity. Moreover, binding affinities can be tuned by optimizing the scaffolds that connect aptamers of multivalent constructs. Thus, the multivalent approach promises to improve binding affinities of aptamers. However, challenges of this approach include the paucity of aptamer pairs that recognize distinct sites of the same target. Accordingly, selection methods that enable isolation of aptamer pairs have been reported, and have extended the range of potential applications of multivalent aptamers.
